# Atomistic Simulations on Metal Rod Penetrating Thin Target at Nanoscale Caused by High-Speed Collision

**DOI:** 10.3390/nano11113160

**Published:** 2021-11-22

**Authors:** Yong-Chao Wu, Jin-Ming Liu, Wei Xie, Qing Yin, Jian-Li Shao

**Affiliations:** 1 State Key Laboratory of Explosion Science and Technology, Beijing Institute of Technology, Beijing 100081, China; yongchao_wu@bit.edu.cn; 2 Defense Engineering Institute Academy of Military Sciences, Beijing 100039, China; Liujm1025@outlook.com (J.-M.L.); xieweixiongqi@163.com (W.X.); 48423348cathy@163.com (Q.Y.); 3 Explosion Protection and Emergency Disposal Technology Engineering Research Center of the Ministry of Education, Beijing 100039, China

**Keywords:** hypervelocity impact, penetration, aluminum, molecular dynamics

## Abstract

The penetration process has attracted increasing attention due to its engineering and scientific value. In this work, we investigate the deformation and damage mechanism about the nanoscale penetration of single-crystal aluminum nanorod with atomistic simulations, where distinct draw ratio (∅) and different incident velocities (u_p_) are considered. The micro deformation processes of no penetration state (within 2 km/s) and complete penetration (above 3 km/s) are both revealed. The high-speed bullet can cause high pressure and temperature at the impacted region, promoting the localized plastic deformation and even solid-liquid phase transformation. It is found that the normalized velocity of nanorod reduces approximately exponentially during penetration (u_p_ < 3 km/s), but its residual velocity linearly increased with initial incident velocity. Moreover, the impact crater is also calculated and the corresponding radius is manifested in the linear increase trend with u_p_ while inversely proportional to the ∅. Interestingly, the uniform fragmentation is observed instead of the intact spallation, attributed to the relatively thin thickness of the target. It is additionally demonstrated that the number of fragments increases with increasing u_p_ and its size distribution shows power law damping nearly. Our findings are expected to provide the atomic insight into the micro penetration phenomena and be helpful to further understand hypervelocity impact related domains.

## 1. Introduction

Hypervelocity collision is a basic problem in the field of high-pressure science and space technology [1], in which high-speed penetration has attracted abundant attention in recent decades. As is known to all, the rigid body approximation can be applied to theoretically analyze the penetration of metallic long rod, which possesses strong capability to the crush and damage of the target, due to corresponding large draw ratio, high density and giant specific kinetic on the unit of section. However, high-speed impact always causes serious geometry deformation, damage and even phase transformation, such as melting and sublimation [2,3,4]. Besides, the penetration mechanism can also be changed with the variation of impact kinetic. The simplified rigid body assumption, thus, is not enough to deepen the understanding of related deformation mechanism and intrinsic physical law. In practice, the hypervelocity impact process is a kind of mechanical, physical and chemical coupling problem [5] involving elastic-plastic deformation, shock waves, heat effect and damage evolution, etc., leading to great difficulty in the traditional experimental and numerical simulation methods [6,7]. It is still challenging to trace the real-time detail of damage and fragmentation process for the state-of-the-art experimental technology due to its tiny spatial and temporal scale and extreme high temperature and pressure ambient.

The macro numerical simulation always involves many empirical or artificial parameters, such as the finite element method, which need to be validated by experiments and cannot directly capture the micro physical properties, but sometimes micro-scale effects play an important role on the micro/nano scale impact process [8,9,10]. For instance, the size effects in porous material can hinder the properties of plastic shock waves based on the theoretical and numerical analysis [8]. Our previous work revealed that the release wave generated from side surface can restrain the continuous growth of voids in the hypervelocity impact of aluminum nanorod and change the spall mode eventually [10]. In summary, revealing microstructure deformation mechanism during hypervelocity impact process is essential for either constructing macro model or application on micro/nano scale design of advanced material. Molecular dynamics (MD) simulation based on Newton’s laws has been a resourceful tool to exploit the various micro properties of materials because it can directly provide the atomic insight into microstructure evolution and the real-time atomic coordinates, forces and energies, etc. Abundant work related to damage or spallation has been successfully conducted using MD simulations [10,11,12,13,14,15]. Many factors, such as the shock intensity [16], loading rate [17], crystalline orientation [18,19], intrinsic defect [20,21] impurity [22] and grain size [23,24], have been considered. 

Except the research mentioned above focused on the one-dimensional shock loading, the complex collision process has also attracted extensive interest in recent years because it involves complex damage mode and abundant physical phenomena [25,26,27,28,29,30,31,32,33]. Experimentally, laser-induced projectile impact tests have shown that multilayer graphene possesses an approximately ten times higher specific penetration energy under an impact velocity of 600 m/s compared with that of macroscopic steel [32]. In terms of simulation, a recent work focused on the graphene/copper nanolayered structure under ballistic impact has shown that graphene interface can change the spall mode and promote the self-healing and recrystallization at the impacted region [29]. Additionally, Dewapriya et al. [26,31,33] carried out a series of MD simulations to investigate the penetration process of polymer/ceramic and polymer/metal, and the results revealed that the composites possess ultrahigh specific penetration energy. However, most of existing collision research adopted rigid bullet and the impact velocity is relatively low, while the plastic deformation in bullet and high incident kinetic may affect the penetration process at nanoscale, such as crater geometry and fragmentation process, the corresponding work is still in its infancy.

The present work puts the emphasis on the deformation mechanism and mechanical properties evolution during the penetration process. A series of MD simulations on the commonly used face-centered cubic aluminum nanorod have been conducted and geometry effects and incident velocity of nanorod were considered. We demonstrate the deformation process of nanorod and nano-target, fragmentation size and crater characteristics. All these results should provide theoretical guidance to understand the fundamental physical mechanism of the micro penetration phenomena. 

## 2. Simulation Details

The classical MD method calculates many-body interaction based on Newton’s laws using interatomic potential, accelerating to simulate large-scale atomistic system. The potential is, thus, the physical basis of accuracy of simulations, which is always, in practice, empirical and optimized to specific target systems using different forms. The embedded-atom method (EAM) was developed based on the density function theory [34], which has been proved to describe the atomic interaction among metal or alloy system suitably. This work adopted the EAM potential developed by Zhakhovskii et al. [35], which can simulate the behavior of aluminum crystal under strong dynamic compression and tension, and has been successfully applied to investigate the hypervelocity impact [10], spallation [36] and crystal melting [37].

**Figure 1:**
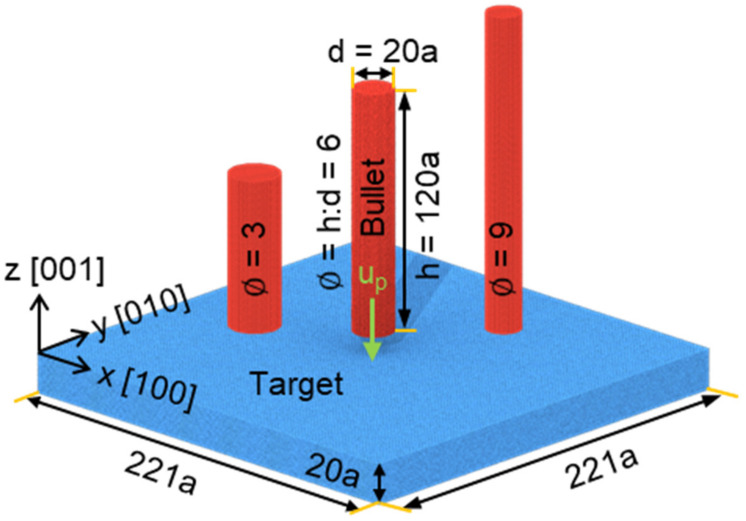
Initial configuration of the target and bullet with different draw ratio. Both the target and bullet are perfect FCC single-crystal aluminum. The atoms are colored by the target (blue) or bullet (red) and represent no physical properties.

Initial configuration is shown in Figure 1. The target part has the length of 221a and thickness of 20a (here, a is the lattice constant and a = 0.405 nm), and the primary directions are orientated as follows: x [100], y [010] and z [001]. The thickness is enough to simulate plastic deformation in the target during impact process, such as dislocation and stacking faults. Three draw ratios (∅=h/d, h and d are respectively the length and diameter of nanorod, ∅=3, 6 and 9) were considered to construct the bullet part, which was located at the plane center of the target and involved about 150,000 atoms equally. The length of the target is more than ten times the diameter of the bullet and able to minimize the boundary effects [26]. Both the target and bullet were perfect face-centered cubic FCC single-crystal aluminum and shared the same crystalline orientation. The system contained more than 4,170,000 atoms.

To obtain stable system structure, the conjugate gradient method was firstly used to minimize the system energy and adjust the atomic coordination. The system then experienced fully relaxation under isothermal-isobaric ensemble (NPT, i.e., constant particle number, pressure and temperature) with a Nosé-Hoover thermostat and barostat [38] for 40 ps until the pressure (0 bar), temperature (300 K) and energy reached an equilibrium state. The impact simulation was then conducted by adding different incident velocities along the z axis (denoted as u_p,_ ranging from 1 to 5 km/s) to the bullet part in microcanonical ensemble (NVE, i.e., constant particle number, volume and energy) for 50 ps. The velocity range has included both unpenetrated and totally penetrated cases from our results. The free boundary was adopted in all three directions during relaxation and impact process. The timestep was selected as 1 fs for all the simulations here, which were realized by open source MD code LAMMPS [39]. The post-processor and visualization of simulation generated atomic trajectory was conducted by OVITO [40]. The microstructure evolution during the penetration process are obtained with the adaptive-CNA method (a modifier embedded in OVITO) [41], based on which the atoms can be recognized as face-centered cubic (FCC), body-centered cubic (BCC), hexagonal close-packed (HCP) and unrecognized (Other) structure.

## 3. Results and Discussion

### 3.1. Characteristics of Penetration at Nanoscale

**Figure 2:**
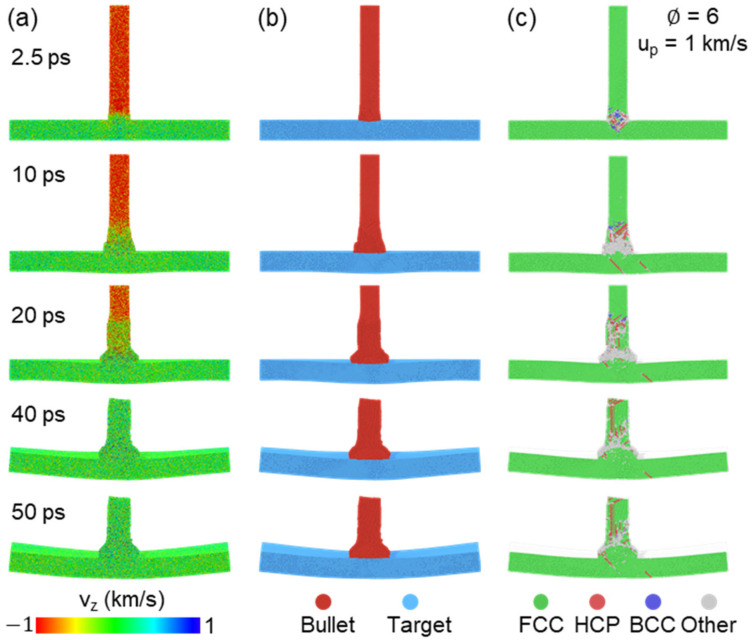
Unpenetrated atomic configurations at several times for the case of ∅=6 and u_p_ = 1 km/s. The atoms are colored by (**a**) velocity along impact direction, (**b**) matter distribution and (**c**) microstructure recognized by adaptive-CNA method.

Penetration state is strongly related to incident kinetic. We selected the case of ∅=6 as an example to elaborate the deformation mechanism. Several snapshots for velocity distribution, matter distribution and microstructure at the collision speed (u_p_) of 1 km/s are shown in Figure 2. Both transmission and reflection shock waves are generated at impact plane at 2.5 ps, as shown in Figure 2a. The impact plane gains velocity equal to about 0.5 u_p_. The three-dimensional shock wave is then generated from the impact center region in the target propagating outside almost spherically. The reflection at the target surface of this shock wave causes the apparent deformation of the target part (see t = 20, 40, 50 ps in Figure 2a). Besides, giant velocity discrepancy between impact center region and outsider region leads to strong plastic deformation near the impact plane, as shown in Figure 2c. For the bullet part, obvious shock wave propagation and upsetting deformation behind the shock wave front is observed, which finally shortens the length of the bullet near 50% compared with initial configuration, while increase the contact area between the bullet and target at 50 ps, as shown in Figure 2b. Many disordered atoms are observed at the impact region, due to the effect of high temperature and high pressure at 10 ps, while those disordered atoms gradually disappear under the effect of reflection and interaction of inside the shock wave at 50 ps, as shown in Figure 2c, which shows the target part has mostly recovered to original regular lattice structure and some residual stack faults are observed in the bullet part. In fact, this kind of self-healing phenomena at nanoscale under shock loading has also been found in previous works [29,42,43], mainly attributed to strong surface/interface effects.

**Figure 3:**
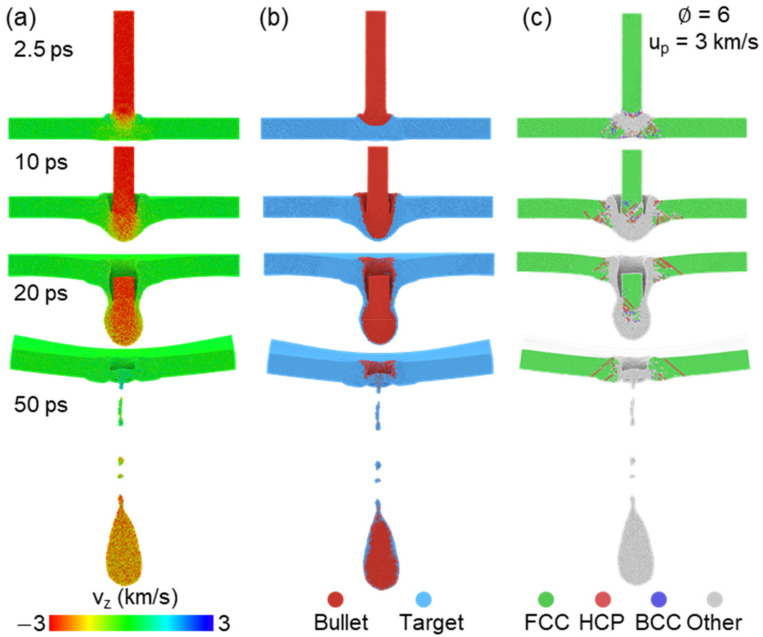
Penetrated atomic configurations at several times for the case of ∅=6 and u_p_ = 3 km/s. The atoms are colored by (**a**) velocity along impact direction, (**b**) matter distribution and (**c**) microstructure recognized by adaptive-CNA method.

Complete penetration state is observed once u_p_ is increased to 3 km/s, and corresponding velocity distribution, matter distribution and microstructure are presented in Figure 3. The bullet maintains a relatively high speed during the entire penetration process, and only a small amount of deformation in the whole target part is found before the bullet completely runs through the target, as shown in Figure 3a. Besides, Figure 3b further indicates that the bullet changes to teardrop-shaped and most of the bullet penetrates the target at 50 ps. Many disordered atoms exist in the impact region at entire penetration process for the target. The end of the bullet keeps near the FCC structure during penetration (such as 20 ps), but it transforms to a disordered state at 50 ps (see Figure 3c), which seems to indicate the local temperature increase and even melting under the effect of release and interaction of the shock wave.

**Figure 4:**
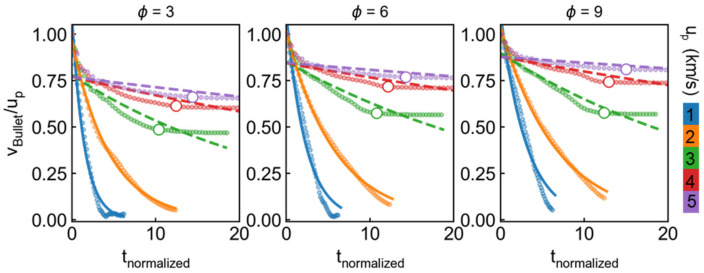
History of bullet velocity v_bullet_ with time under different u_p_ and ∅. Both axes are normalized to simplify analysis. X-axis is normalized by the form of: $t_{\mathrm{normalized}}=t\times u_{p}/t_{t}$, where t_t_ indicates thickness of target and is equal to 8.1 nm. The solid points are original data and hollow points represent the inflection points of curve. The solid line and dash line are the fitted data by the form of: $y={\mathrm{ae}}^{\mathrm{bx}}$, where a and b are two fitted parameters.

To further understand the details of the penetration process, the time evolution of average bullet velocity v_bullet_ has been calculated. Figure 4 presents the evolution of normalized v_bullet_ changing with normalized time t_normalized_ under different initial incident velocity and distinct draw ratio. Normalized x-axis is defined for simplifying analysis and counted by the form of: $t_{\mathrm{normalized}}=t\times u_{p}/t_{t}$, where t_t_ indicates the thickness of the target and is equal to 20a, that is 8.1 nm. For the cases of u_p_ > 2 km/s, the bullet velocity experiences rapid attenuation before penetration then transforms to a relatively slow decrease for all the draw ratios, the former can be attributed by yield behavior of target and the latter appears to result from the softening effect of the material. In contrast to high-speed impact, the bullet velocity directly decreased to near zero at t_normalized_ = 10 as u_p_ ≤ 2 km/s (not penetrated). We use exponential function to describe such attenuation trend by the form of: $y={\mathrm{ae}}^{\mathrm{bx}}$, where a and b are two fitted parameters. It is found that the case of low velocity (u_p_ ≤ 2 km/s) can be well described by exponential function, as marked by solid line in Figure 4, while relatively large discrepancy is observed at high-speed impact (see dash line in Figure 4). It is noting that the trend of velocity variation is similar for different draw ratio ∅ we considered, while the residual velocity increases with increasing ∅, which will be further discussed in the following contents.

**Figure 5:**
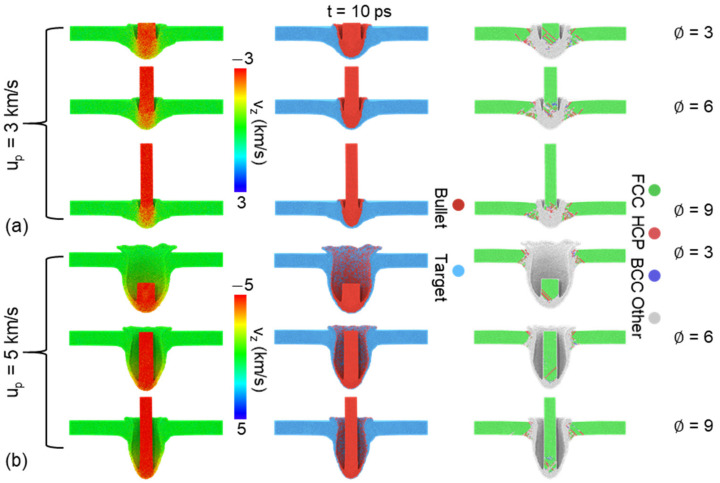
Atomic configurations at 10 ps for different ∅ at the case of (**a**) u_p_ = 3 km/s and (**b**) u_p_ = 5 km/s. Atoms are colored by velocity along impact direction (first column), matter distribution (second column) and microstructure recognized by adaptive-CNA method (third column).

Figure 5 compares the characteristics of penetration with different ∅ at u_p_ = 3 and 5 km/s. The impact front of the bullet forms similar spike under relatively low velocity (3 km/s). For the case of ∅=3, over half of the bullet mixes with the target and causes large damage area compared with the case of ∅=9, which possesses smaller contact region, as shown in Figure 5a. Noting that the radius of crater is close to the radius of the bullet at the moment. However, due to the strong release effect at the bottom surface of the target after strong loading, the damage mode is not limited to localized amorphization, but transformed to uniform spherically fragmentation at the high-speed impact (5 km/s), as shown in Figure 5b. Obviously, higher incident kinetic can form larger damage area and produce more fragmentations.

**Figure 6:**
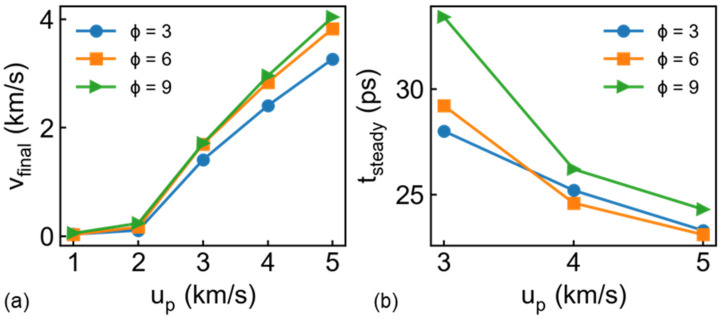
Relation between u_p_ and (**a**) bullet velocity at 50 ps v_final_ and (**b**) penetration time t_steady_, which is defined by the inflection point in bullet velocity history.

The final residual bullet velocity v_final_ and penetration time for different ∅ at different u_p_ are presented in Figure 6. Firstly, the penetration performance of different materials can be compared by a commonly used parameter, that is, ballistic limit velocity, which is defined as the lowest velocity required to penetrate the target completely. Here, the ballistic limit velocity can be roughly treated as 3 km/s for all the draw ratio, as shown in Figure 6. We found that v_final_ maintains linear increase relation with u_p_ for the bullet with different ∅ (u_p_ ≥ 3 km/s). Besides, obvious increase of residual velocity exhibits increasing ∅ from 3 to 6, while this trend becomes unclear with further increasing ∅ from 6 to 9, appearing to imply a limited value for draw ratio in the penetration process. That means complete penetration and subsequent inertia-driven motion. In this case, we further present the function of penetration time and incident velocity in Figure 6b. Obviously, penetration time decreases with increasing u_p_, especially for the case of high draw ratio. Noting that the thinnest bullet (∅=9) experiences the longest penetration time related to the apparent geometric dimension.

**Figure 7:**
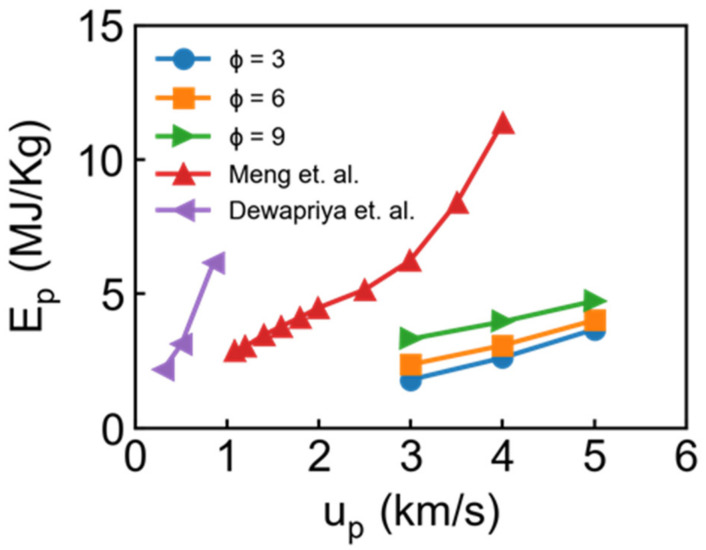
Relation between u_p_ and specific penetration energy $E_{p}$ for different draw ratio. The red points and purple points come from works reported by Meng et al. [30] and Dewapriya et al. [31], respectively.

Based on the final velocity in Figure 6, we can obtain the kinetic energy loss $\Delta {\mathrm{KE}}_{b}$ of the bullet by the form of: $\Delta {\mathrm{KE}}_{b}=0.5\left(m_{b}u_{p}^{2}-m_{b}v_{\mathrm{final}}^{2}\right)$, where $m_{b}$ is the mass of bullet. However, normalized $\Delta {\mathrm{KE}}_{b}$ is essential to compare with other material quantitatively because initial $\Delta {\mathrm{KE}}_{b}$ is highly dependent on the thickness of the target and size of the bullet [30]. The corresponding specific penetration energy $E_{p}$ can be calculated by the form of: $E_{p}=\Delta KE_{b}/\left(\rho_{t}A_{s}t_{t}\right)$, where $\rho_{t}$ and $t_{t}$ are the density and thickness of the target, and $A_{s}$ is the strike face area in the target. Generally speaking, $A_{s}$ is approximately calculated from: $A_{s}={\pi R}_{b}^{2}$, where $R_{b}$ is the radius of the bullet because the crater radius is close to the bullet radius at the case of thin target thickness and little rigid bullet [30]. However, the aluminum bullet in present work can cause a large impact region. Hence, we adopt $A_{s}={\pi R}_{c}^{2}$ to calculate the impact area, where $R_{c}$ is the radius of the crater (detailed discussion can be seen in Section 3.2). This assumption may change the absolute value of $E_{p}$, but we aim to describe the variation trend. The specific penetration energy is compared in the Figure 7. Obviously, $E_{p}$ increases with increasing incident velocity and draw ratio, whose trend is consistent with results from [30] (2 nm thickness aluminum slab and rigid spherical bullet) and [31]. However, we found the $E_{p}$ from [30] is over 2.5 times higher than our results, which appears to be attributed to the effect of the thickness of the target and characteristic of the bullet. In other words, our model is not suitable to calculate accurate specific penetration energy with a softer bullet compared with a rigid bullet, but further investigation is out of scope of this work.

**Figure 8:**
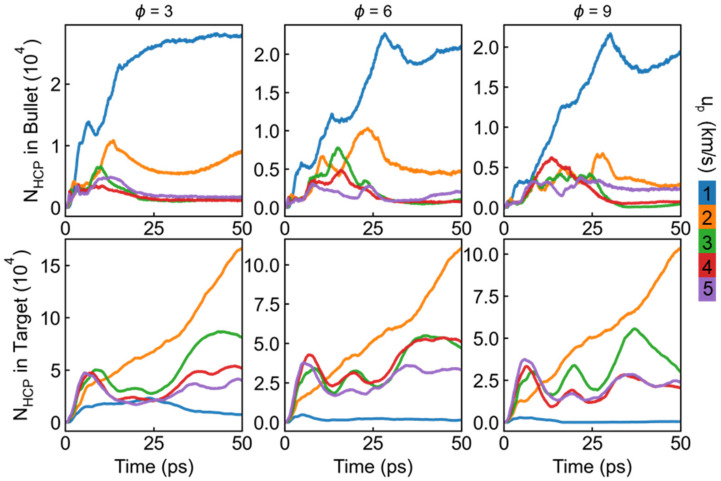
History of the number of HCP atoms N_hcp_ in bullet and target for different draw ratio and incident velocity.

To roundly understand the elastic-plastic deformation mechanism in impacted material, the number of HCP (stacking faults) atoms N_hcp_ in both the bullet and target for different ∅ at different u_p_ are summarized in Figure 8. For the bullet part, the stacking fault and slip dominate the plastic deformation behavior at the case of low incident velocity (1 km/s), and the HCP atoms number, thus, increases rapidly from the beginning of the impact process, while obviously decreases when the u_p_ increases to 2 km/s. It is found that the HCP atoms number in the bullet shows minor discrepancy as the u_p_ higher than 3 km/s, indicating the main deformation mechanism has transformed to amorphization and melting at such high incident kinetic. With regard to the target, the number of HCP atoms at the case of 2 km/s is larger than other incident velocity, which can be attributed to strong nanoscale surface effect. Specifically, the incident kinetic is not high enough to penetrate the target completely and hence mainly acted as a deformation energy source, resulting in remarkable overall deformation in the target. For the cases of u_p_ ≥ 3 km/s, a similar variation trend is found in the target. The draw ratio of the bullet also plays an important role in the history of HCP atoms number. The maximum of HCP atoms number decreases with the increasing ∅ in both the target and bullet at the case of u_p_ ≤ 2, while it exhibits similar variation range at the higher incident velocity.

### 3.2. Crater and Fragematation Process

An impact-induced crater is a significant phenomenon in the penetration process, and its geometry characteristics can be approximately described by depth and radius. Considering the small thickness of the target in this work, penetration depth can be simply thought to be equal to the thickness of the target. The emphasis here is put on the definition of the radius of the crater though it is hard to accurately describe the real radius of a unregular crater surface. Here we propose a procedure to obtain the equivalent radius: Step one: define a number of connected atoms within the cutoff distance (r_c_, here r_c_ is selected equal to nearest neighbor distance, i.e., 0.286 nm) as a cluster, then the atoms in the bullet and in the target within r_c_ can be distinguished from the impact region, which is thought as crater surface; Step two: the highest 1000 atoms along the impact direction (z-axis) are selected as reference points, and the geometry center of those atoms can be set as the center of a circle; Step three: a series of gradually increasing circles with a step length of 0.3 nm (an empirical parameter) are generated, once a circular ring includes more than 50 atoms (an empirical parameter), the present radius can be treated as the equivalent radius of the crater.

**Figure 9:**
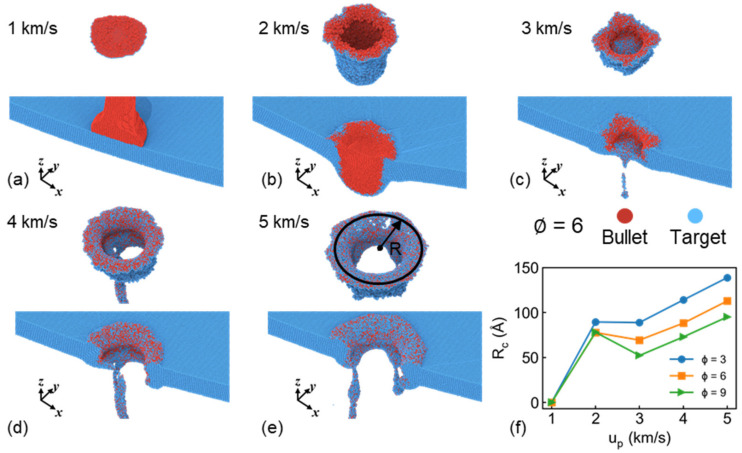
Crater surface and cross-section of sample at 50 ps under u_p_ of (**a**) 1 km/s, (**b**) 2 km/s, (**c**) 3 km/s, (**d**) 4 km/s and (**e**) 5 km/s at the case of ∅ = 6; (**f**) Radius of crater $R_{c}$ under different u_p_ and draw ratio of bullet. Atoms are colored by matter distribution.

Based on the above procedure, the radius of the crater $R_{c}$ and corresponding crater surface at 50 ps are presented in Figure 9. No obvious crater is produced at the case of 1 km/s, where the bullet mixes with the target surface finally. For the case of 2 km/s, the target is not penetrated completely, though forms a clear crater. With increasing incident velocity, the complete penetration is found. The radius shows linear increase with incident velocity at such cases, while decreases with increasing draw ratio, as shown in Figure 9f, which is consistent with the microstructure results in Figure 5. Interestingly, we noticed that the crater radius decreases from 2 to 3 km/s at the case of ∅ = 6 and 9 because the bullet has not fully penetrated the target at the case of 2 km/s, and thus the incident kinetic energy mainly contributes to plastic deformation or partial melt at the impact region, which leads to larger bumps of crater. As incident velocity increases to 3 km/s, its kinetic energy is consumed by penetration along impact direction and the transverse expansion is relatively small. The crater surface can be seen in Figure 9b,c, indicating the reasonability of our proposed procedure.

**Figure 10:**
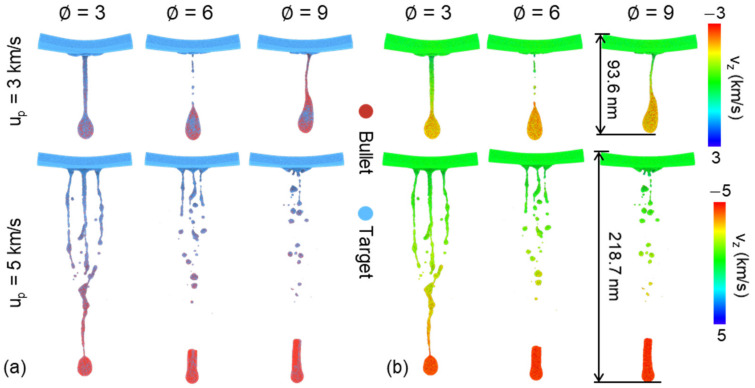
Microscopic views of final fragmentation (50 ps) for different draw ratio at the case of 3 and 5 km/s. Atoms are colored by (**a**) matter distribution and (**b**) velocity along impact direction.

Fragmentation after penetration is of concern because it can help understand the material shock response. This kind of phenomena can be always observed in the high-speed velocity impact field, such as micro-ejecta [44], which occurs when the plane shock wave propagates through a material-vacuum interface and a mass of small fragmentations are emitted from the material surface. The characteristic of fragmentation is related to shock intensity and surface geometry. Another case is impact-induced fragmentation, the high local temperature leads to solid-liquid phase transformation and the intrinsic velocity gradient causes final separation and develops to fragmentation [10]. Spatial distribution and geometry of fragmentation has presented in Figure 10 for the case of 3 and 5 km/s. When incident velocity is relatively low (3 km/s), the bullet maintains connected with the target at final state (50 ps) and a small number of fragmentations was produced, mainly including a teardrop-shaped debris, mixed by the bullet and target, as shown in Figure 10a. The draw ratio shows minor effects on the characteristics of fragmentation. However, the bullet with high incident velocity (5 km/s) can rapidly penetrate the target and cause more fragmentation, involving an obvious residual end of the bullet and many nearly spherical metallic clusters from the target (due to surface tension). Besides, draw ratio of the bullet can influence the size and number distribution of fragmentation. The microscopic views indicate the fragmentation is close to the ribbon at the case of ∅ = 3, while transformed to nearly spherical shape at the case of ∅ = 6 and 9, as shown in Figure 10b. Noting that the fragmentation also shows crystallographic dependence at the case of ∅ = 3 and u_p_ = 5 km/s, which has also been observed in previous work [10].

**Figure 11:**
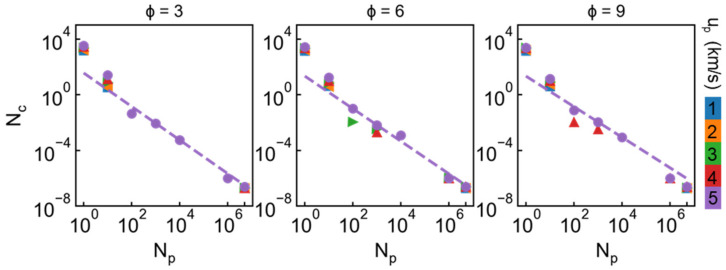
Number and size of cluster for incident velocity ranging from 1 to 5 km/s under different draw ratio. N_p_ indicates the atom number in each cluster and N_c_ means the number probability of corresponding N_p_ range. Dash line is the power law fitting data for the case of 5 km/s, ignoring the size lower than 1000 atoms.

The number and size of cluster are further estimated based on the method used in works [10,44,45], as shown in Figure 11. N_p_ indicates the atom number in each cluster and N_c_ means the number probability in corresponding N_p_ range. The number and size distribution exhibit similar features for all the cases. Previous works have shown the fragmentation distribution approximately manifests the power law decrease regulation in micro-ejecta or nanorod impact [10,44,45]. Here we use similar formula to fit original data: $N_{c}={\mathrm{aN}}_{p}^{b}$, where a and b are undetermined parameters. We take the case of 5 km/s as original data because more fragmentation is formed at this incident velocity. Besides, N_p_ < 1000 is not considered for the fitting, which is regarded as insignificant in the fragmentation process. The fitting results show that the fragmentation distribution in the penetration process also manifests this attenuation trend, as marked by dash line in Figure 11. Those results appear to reveal a universal regulation for fragmentation distribution under hypervelocity impact at nanoscale.

## 4. Conclusions

The penetration process of nanorod under different incident velocity and draw ratio has been investigated based on a mass of large-scale atomistic simulations. The penetration threshold value for all draw ratios is 2 km/s. Stacking fault and slip dominate the plastic deformation when u_p_ ≤ 2 km/s, otherwise the amorphization and melting explain mainly deformation behavior. The bullet velocity decreases with exponential trend at relatively low initial velocity (≤2 km/s), while the final residual velocity increases linearly with increasing initial velocity. The draw ratio exerts minor effect on the evolution trend of stacking faults while obviously influences the variation degree at relatively low velocity. The radius of impact-induced crater shows linear increase in relation to initial velocity at the penetrated stage (>2 km/s). Besides, the geometry and size distribution also analyzed, and the latter manifests power law decrease regulation. Our findings are helpful to construct the theoretical model for nanoscale penetration and hypervelocity impact related fields.

## Data Availability

The data presented in this study are available on request from the corresponding author.
